# A Novel Treatment of Acne Fulminans with Adalimumab: A Case Report

**DOI:** 10.51894/001c.7003

**Published:** 2018-09-26

**Authors:** Roxanne Rajaii, Jeff Globerson, Nichelle Arnold, Michael Mahon

**Affiliations:** 1 Department of Dermatology, Farmington Hills, Michigan Beaumont Health Systems

**Keywords:** tnf-alpha inhibitor, humira, adalimumab, acne fulminans

## Abstract

Acne fulminans (AF) is a rare and highly inflammatory severe form of acne most commonly seen in adolescent males. Unlike acne vulgaris, AF presents with associated systemic manifestations including, but not limited to, malaise, myalgia, arthralgia, fever, anorexia, and weight loss. It is often an extremely painful condition of sudden onset and can occur years after mild or moderate acne vulgaris. While the inciting agent for this condition has been postulated to be an explosive hypersensitivity reaction to the bacterium Propionobacterium acnes, increased androgens, namely testosterone, have also been reported to play a role in the pathogenesis of this disease process. Additionally, environmental triggers such as air pollution and exposure to halogenated hydrocarbons during occupational activities in enclosed, high temperature settings have been identified as possible etiologies or exacerbating factors. AF is primarily a clinical diagnosis. Isotretinoin, in combination with systemic steroids, are generally the treatments of choice for this disease entity. A Caucasian male in his early 40’s presented to the authors’ clinic with a chief complaint of painful acneiform nodules, cysts, papules, pustules, and abscesses on his back, chest, neck, shoulders, upper arms, and thighs for several months. This case report demonstrates a refractory case of AF with significant clinical improvement after six weeks of topical treatment with subcutaneous adalimumab in combination with oral doxycycline. This case provides evidence supporting the role of Adalimumab in the treatment of AF in addition to the other inflammatory conditions currently FDA approved for treatment with this tumor-necrosis factor (TNF) alpha inhibitor. These conditions include plaque psoriasis, Crohn’s disease, hidradenitis suppurativa, psoriatic arthritis, and rheumatoid arthritis.

## INTRODUCTION

Acne fulminans (AF) is a rare, highly inflammatory and severe form of acne presenting with an eruption of painful nodules, pustules, and hemorrhagic ulcerations in association with systemic manifestations such as fever, arthralgia, myalgia, hepatosplenomegaly, leukocytosis, anemia, and increased inflammatory lab markers including TNF-alpha.^1^ This painful condition usually presents itself in young adolescent males with a previous history of moderate acne vulgaris. The pathogenesis appears to be multifactorial.^1,2^

AF is primarily a clinical diagnosis and treatment is often challenging at best. To date, combination therapy with isotretinoin and systemic corticosteroids have been the treatments of choice.^1,3–7^ Novel studies using tumor-necrosis factor alpha (TNF-alpha) inhibitors have shown great promise for recalcitrant inflammatory conditions such as plaque psoriasis, Crohn’s disease, hidradenitis suppurativa, (i.e., inflammatory skin disease that affects apocrine gland bearing skin in axillae, groin, etc.) psoriatic arthritis, and rheumatoid arthritis in various medical settings.^7^ The markedly inflammatory nature of AF and success of TNF-alpha inhibitors in treating other chronic inflammatory conditions suggests their possible applicability in the management of AF.

## METHODS

### Case Report:

A Caucasian male in his early 40’s presented to the authors’ Dermatology clinic with a chief complaint of painful acneiform nodules, cysts, papules, pustules, and abscesses on his back, chest, neck, shoulders, upper arms, and thighs for several months. He reported a history of mild acne throughout his adolescence and early adulthood prior to this eruption. His associated symptoms included tenderness, swelling, pain, and drainage in the areas affected by these acneiform papulonodules and abscesses, most notably on the back, chest, and shoulders.

He reported constitutional symptoms of arthritis and occasional fevers but denied chills, night sweats, myalgia, malaise, and weight loss. He had failed previous treatment with multiple oral and topical antibiotics over a course of approximately one year, isotretinoin therapy for roughly six months, and phototherapy, oral/intralesional steroids, and incision/drainage. Each of these treatments had been delivered on an as-needed basis.

On examination, this patient demonstrated widespread erythematous (i.e., reddened) and inflamed acneiform nodules, cysts, papules, pustules, and abscesses on his back, chest, neck, shoulders, upper arms, and thighs. (Figure 1) Several cysts were fluctuant (i.e., compressible) and tender with active serosanguineous (i.e., containing both blood and serum) drainage. On further exam, he had widespread ill-defined hyperpigmented patches. The worst areas of involvement included the trunk, namely the back.

**Figure:**
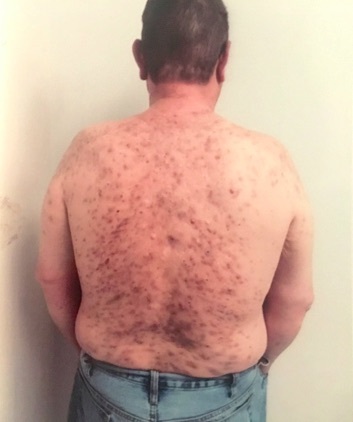
Figure 1 Acne Fulminans Presenting with Widespread Erythematous and Inflamed Acneiform Nodules, Cysts, Papules, Pustules, and Abscesses on the Back.

## RESULTS

This man was diagnosed with AF and started on a treatment regimen of topical and oral medications including topical clindamycin and Tretinoin, once weekly phototherapy, Oral medications included Doxycycline 100 mg twice daily, isotretinoin, and oral and intralesional steroids, all with minimal improvement after a total of 12 months.

Due to his limited multi-therapy success, and after appropriate preliminary laboratory evaluation, he was started on the following subsequent regimen: continuation of Doxycycline and initiation of self-administered subcutaneous injections with 40 mg of adalimumab every two weeks. After only six weeks of treatment on this new regimen, he exhibited a significant reduction in inflammatory nodules, cysts, papules, pustules, and abscesses. (Figure 2)

**Figure:**
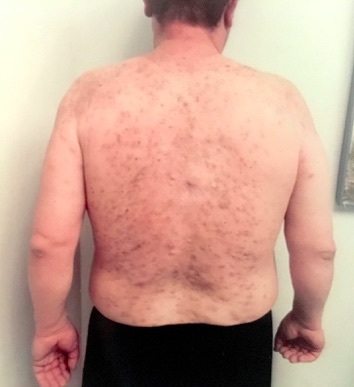
Figure 2 Acne Fulminans after Six Weeks of Treatment on Combination Therapy with Doxycycline and Adalimumab. A Significant Reduction in Inflammatory Nodules, Cysts, Papules, Pustules, and Abscesses Was Seen on the Back.

## DISCUSSION

AF is a rare and severe form of inflammatory acne characterized by the abrupt onset of painful nodules, pustules, and hemorrhagic ulcerations in association with systemic manifestations including fever, arthralgia, myalgia, hepatosplenomegaly, leukocytosis, anemia, and increased inflammatory lab markers such as TNF-alpha levels.

Additionally, osteolytic bone lesions (i.e., “punched-out” areas of severe bone loss) may be detected, most commonly involving the clavicle and sternum.^1^ AF primarily affects the face, neck, arms, back and chest of adolescent boys age 13-16 with a previous history of mild or moderate acne vulgaris.^3^ The pathogenesis appears to be multifactorial, and it is postulated that the condition is prompted by an explosive hypersensitivity reaction to *Propionobacterium acnes*, increased androgens, namely testosterone, and environmental triggers such as air pollution and exposure to halogenated hydrocarbons in occupational high temperature settings which promote occlusion and excess sweating.^1,2^

Although various options have been described, the treatment of AF remains a challenge for clinicians. The current recommendations suggest a combination treatment with systemic oral prednisolone and oral isotretinoin.^4^ Suekeran and Cunliffe’s 1999 review of 25 cases recommended 0.5 - 1.0 mg/kg oral prednisolone daily for 4-6 weeks with the addition of oral isotretinoin therapy starting in the fourth week at a dose titrated up from 0.5 mg/kg daily.^4^ The total duration of treatment has been variable and in one review ranged from nine months to two years.^1^

Conventional treatments with oral antibiotics, topical corticosteroids, and nonsteroidal anti-inflammatory drugs have been used without significant improvement or sustained remissions.^3^ Although effective management can be challenging, recurrent AF has been rarely reported.^4^ The markedly inflammatory nature of AF and success of TNF-alpha inhibitors in treating other chronic inflammatory skin disorders such as plaque psoriasis and hidradenitis suppurativa has led dermatologists to prescribe these medications off-label (i.e., for indications yet to be approved by federal officials) for resistant acne.

Since the mid-1990s to early 2000s, a more complete understanding of immunology and advances in biologic drug development has popularized the use of TNF-alpha inhibitor therapy. Adalimumab is a recombinant human monoclonal antibody that binds to TNF-alpha thereby halting the progression of the cytokine driven inflammatory cascade. Recent reports of TNF-alpha inhibitor therapy for severe inflammatory acne postulate their use and efficacy in patients with AF and provide the impetus for the use of subcutaneous adalimumab in our patient with refractory AF.^6,7^

Early reports of TNF-alpha inhibitor use to control AF lesions involved patients with SAPHO syndrome (Synovitis, Acne, Pustulosis, Hyperostosis, and Osteitis) who had failed conventional treatments.^3^ SAPHO syndrome is a rare chronic inflammatory disease of unknown etiology that may accompany AF and other cutaneous manifestations including acne conglobate (AC), acneiform folliculitis, HS, psoriasis, Sweet syndrome, and pyoderma gangrenosum.

It has been well established that patients with SAPHO syndrome have elevated levels of TNF-alpha from purified neutrophils as well as in bone biopsies analyzed with in situ hybridization and immunohistochemistry.^3^ Treatment of these patients with infliximab and etanercept has demonstrated reductions in neutrophil TNF-alpha, resolution of acneiform lesions, and durable responses.^5^ These results suggest that components of SAPHO syndrome including AF result from an abnormal immunological response to Propionobacterium acnes, and that TNF-alpha inhibitors may be indicated for certain cases of recalcitrant acne and AF.

Recently, TNF-alpha inhibitors adalimumab, etanercept, and infliximab have been used to treat resistant AC, a variant of severe inflammatory acne and part of the follicular occlusion tetrad (i.e., AC, hidradenitis suppurativa, dissecting cellulitis and pilonidal disease). Two case reports describe patients responding to adalimumab monotherapy after failing conventional treatments for recalcitrant AC.^6,7^ After four weeks of treatment with adalimumab, most of the inflammatory lesions had resolved, and after 12 weeks all of the nodular lesions had cleared entirely in both case report patients. No adverse events or abnormal laboratory tests were reported in these cases.^6,7^

Additionally, one patient with AC of the face, neck, and trunk responded to treatment with etanercept demonstrating complete resolution of acneiform lesions at 24 weeks.^8^ In 2006, Shirikawa presented a patient with concomitant rheumatoid arthritis and AC who failed isotretinoin due to adverse effects. Infliximab therapy was initiated, and a rapid reduction in the number and size of cystic lesions was appreciated.^9^ The literature and our case report suggest that TNF-alpha inhibitors can provide clinicians with a reliable and efficacious treatment for patients suffering from severe inflammatory acne refractory to other treatment modalities.

## CONCLUSIONS

The results published in the recent literature support the mechanism and role of TNF-alpha inhibitors in the treatment of recalcitrant inflammatory acne. Our case report further supports these results and provides additional evidence for a role of TNF-alpha inhibitors in the treatment of such resistant inflammatory disorders including AF. While the evidence appears promising, current recommendations for treatment of AF remains a combination of oral isotretinoin and systemic steroids.

Further research in this area is needed to elucidate the efficacy and most appropriate use of TNF-alpha inhibitors in the setting of inflammatory acne, particularly cases that have failed the current standard of care.

### Conflict of Interest

The authors declare no conflict of interest.
